# Mechanical assessment of tripled hamstring tendon graft when using suspensory fixation for cruciate ligament reconstruction

**DOI:** 10.1186/s40634-018-0163-3

**Published:** 2018-11-27

**Authors:** I. Geethan, K. Santhosh Sahanand, P. R. Ashwin Vijay, David V. Rajan

**Affiliations:** 1 Arthroscopy Centre, Trichy, GastroCare Hospital, 11th Cross East, Thillai Nagar, Thiruchirappalli, Tamil Nadu India; 2 Ortho One Orthopaedic Speciality Center, Coimbatore, Tamil Nadu India

**Keywords:** Tripled hamstring tendon, Cruciate ligament reconstruction, Mechanical assessment, Suspensory fixation

## Abstract

**Background:**

Tripling semitendinosus tendon for ACL graft preparation facilitates creation of longer and thicker grafts. Our objective was to evaluate the mechanical difference between tripled tendon grafts, prepared by three methods, by comparing with quadrupled tendon graft.

**Methods:**

Bovine hind-foot hoof extensors were allocated to four groups. Group I had quadrupled graft construct. Tripled graft constructs were prepared by passing the tendon to the Endobutton CL loop and stitching the third strand to (i) the loop (in Group II) or (ii) to one strand(in Group III) or (iii)to loop and both tendon strands (in Group IV). The constructs were preloaded from 10 to 50 N at 0.1 Hz for 10 cycles, followed by 1000 cycles of sinusoidal loading between 50 and 250 N at a frequency of 0.5 Hz. The specimens were then subjected to load to failure test at the rate of 50 mm/min. Displacement with cyclic loading, load at failure and the mode of failure were noted.

**Results:**

The load at failure was 957 ± 23.30 N (Mean ± Standard Deviation) in Group I, 590.8 ± 24.40 N in Group II, 682.6 ± 59.28 N in Group III and 963.4 ± 21.72 N in Group IV. The displacement with cyclic loading was 1.13 ± 0.11 mm in Group I, 4.908 ± 0.55 mm in Group II, 1.822 ± 0.55 mm in Group III and 1. 126 ± .018 mm in Group IV. There was no significant difference between the Groups I and IV with respect to the load at failure and displacement (*p* > 0.05). The values were significantly different in Group II and Group III (*p* < 0.01), when compared to groups I and IV.

**Conclusions:**

Tripled grafts have mechanical properties equivalent to quadrupled grafts only when the three strands are sutured together. Caution may be warranted when using suspensory fixation device with tripled tendons and the third strand must be securely attached to the loop of fixation device and to the other two strands.

## Background

**Fig. 1:**
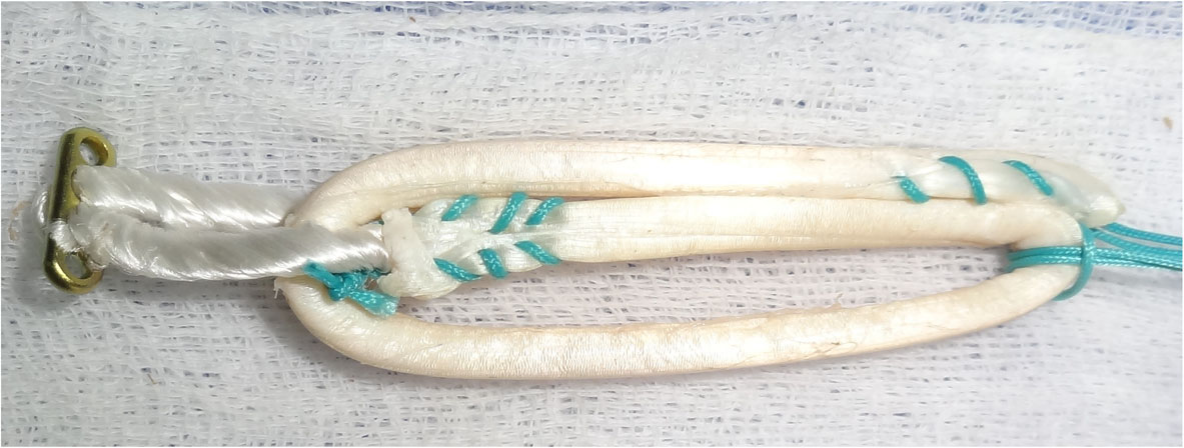
Tripled graft configuration in Group II

**Fig. 2:**
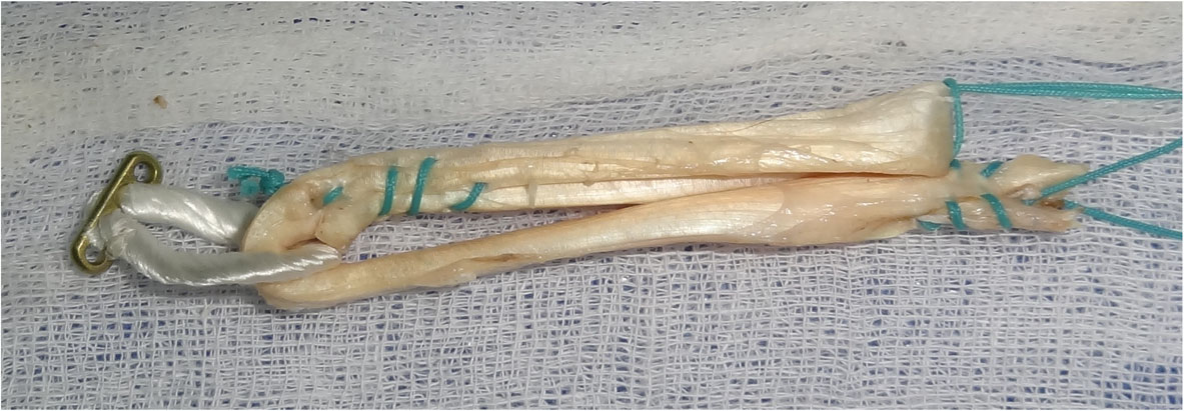
Tripled graft configuration in Group III

**Fig. 3:**
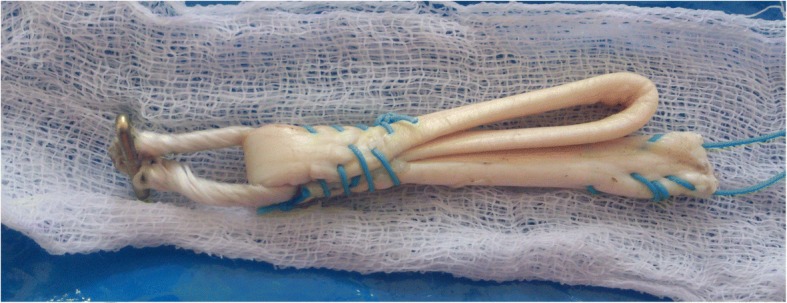
Tripled graft configuration in Group IV

Anterior Cruciate Ligament (ACL) Reconstruction is the standard of care for patients who experience instability due to ruptured ACL. Semitendinosous and gracilis tendon has been one of the preferred graft choices and suspensory fixation devices like Endobutton CL (Smith & Nephew Inc., Andover, Massachusetts) has gained popularity as fixation device (Chechik et al. 2013). Traditionally, hamstring tendons have been used as four strand grafts, either as doubled semitendinosous and gracilis tendons (Ferretti et al. 2011) or as quadrupled semitendinosous tendon (Streich et al. 2013) graft. Subsequently, semitendinosous tendon has been used in triplicate (Chambat et al. 2013; Vinagre et al. 2017; Zysk et al. 2000). Tripled tendon is longer than quadrupled tendon, and is thicker than a doubled tendon. While performing double bundle ACL reconstruction, only one tendon is available for each bundle and tripled grafts will result in grafts of good thickness without compromising on length. Tripling semitendinosous tendon allows preparation of five strand graft, a useful technique to increase graft diameter (Krishna et al. 2018). Thicker grafts are associated with lower meniscal stress, decreased joint laxity, and less articular cartilage contact stress (Westermann et al. 2013). The exact graft diameter needed to avoid failures is not absolutely clear but recent studies suggest than even increases of 0.5 mm up to a graft size of 10 mm are beneficial for the patient, by reducing risk of failure (Magnussen et al. 2012; Snaebjörnsson et al. 2017) and improving function (Mariscalco et al. 2013). While performing single bundle ACL reconstruction, the use of tripled tendons will result in grafts of sufficient length and diameter, potentially sparing gracilis tendon and limiting graft side morbidity (Gobbi 2010). Tripled tendon consists of a doubled portion of tendon, with another strand, the central strand sutured to either the loop of suspensory device or the other strands (Figs. 1, 2 and 3). Failure to suitably incorporate the central strand may result in weaker graft construct. Since suture fixation is an inferior form of fixation, the biomechanical strength of a three strand graft attached to a suspensory fixation device is equivocal (Snow et al. 2012). Moreover, different methods of tripling the tendon graft has been described in literature (Krishna et al. 2018; Lee 2013; Snow et al. 2012; Vinagre et al. 2017; Zysk et al. 2000).

The purpose of this study was to compare the mechanical properties of three different methods of preparing tripled tendon grafts when using suspensory fixation device, comparing with quadrupled tendon graft. Our hypothesis was that tripled tendon grafts prepared by different methods will have variable load to failure and displacement properties when compared to quadrupled tendon grafts of same diameter.

## Methods

This is a controlled laboratory study. Bovine hind limb hoof extensor tendons were obtained from local slaughter house and used within an hour. Tendons were not frozen or thawed. Any tendon showing gross appearance of injury was excluded. All tendons that were macroscopically normal in appearance were included. The tendons were cleared of soft tissues using blunt edge of surgical knife and sized. The tendons that were minimum 320 mm long where allocated to Group I, and those tendons that were minimum 250 mm long were randomly allocated to three other groups by simple randomisation. Endobutton CL fixation button with 20 mm Continuous Loop Suture (Smith & Nephew Inc., Andover, Massachusetts) was used in all samples.

### Placement of whipstitch

**Fig. 4:**
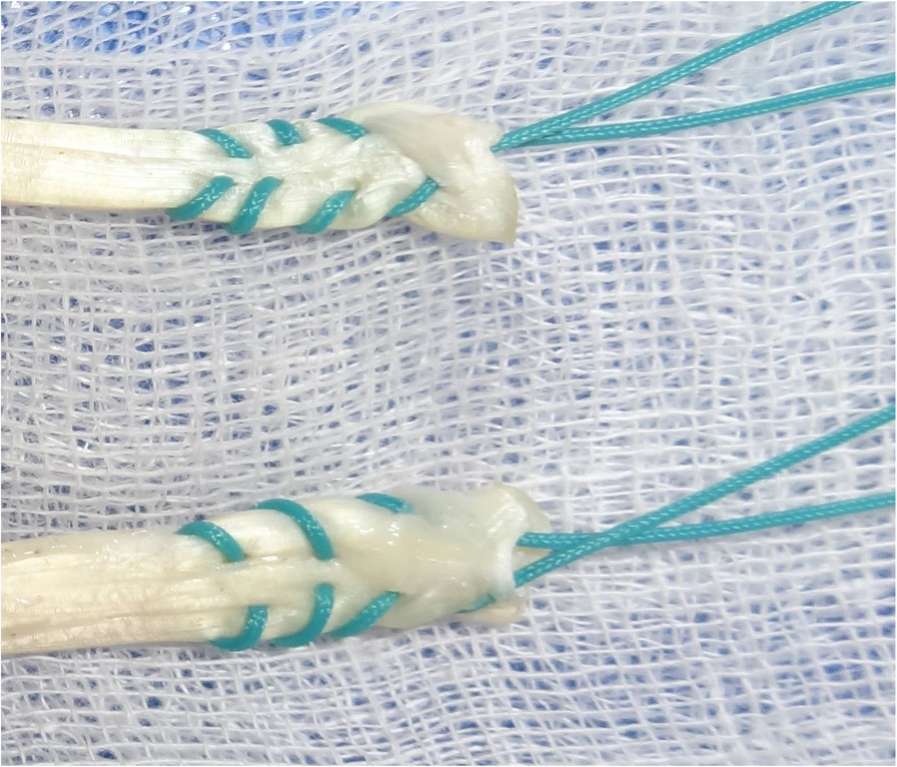
Whipstitch

In all groups whipstitches were placed using a No 2 Ethibond (Ethicon, Somerville, NJ) in the standard fashion (Fig. 4) and were tightened by a strong sustained pull.

### Group I

The tendon was cut into two halves of 160 mm each. All four ends were individually whip-stitched using a No 2 Ethibond as described previously. Both strands were passed through the continuous loop of an Endobutton CL (Smith & Nephew Inc., Andover, Massachusetts) to create a quadrupled graft.

### Group II

The graft was prepared as described by Snow et al. (Snow et al. 2012). Briefly, the tendon was cut to be 240 mm long and the two ends of the tendon were whip-stitched as described previously. The tendon was measured and marked to identify three equal segments. One third of the tendon was passed through the Endobutton CL loop and the remaining two thirds was folded onto itself and the free end with whip stitch was secured to the Endobutton CL loop with a surgical knot consisting of four square knots. Luggage tag stitch was placed on the folded end to facilitate graft holding (Fig. 1).

### Group III

The tendon was cut to be 240 mm and whip stitch was placed one end of the tendon. The whip stitched end of the tendon was passed through the CL loop. The remaining portion of the tendon was folded as in Group II and the free end was sutured onto one limb of the tendon in the loop region (Fig. 2).

### Group IV

Graft was prepared as in Group II and, additionally, all three strands were sutured together (Fig. 3).

All grafts were 80 mm long. Their diameter was measured using a graft sizer (Smith & Nephew Inc., Andover, Massachusetts). Only those grafts that were 8 mm in diameter were chosen for this study. Any sample that was not confirming to these standards was discarded. Five samples were included in each group.

**Fig. 5:**
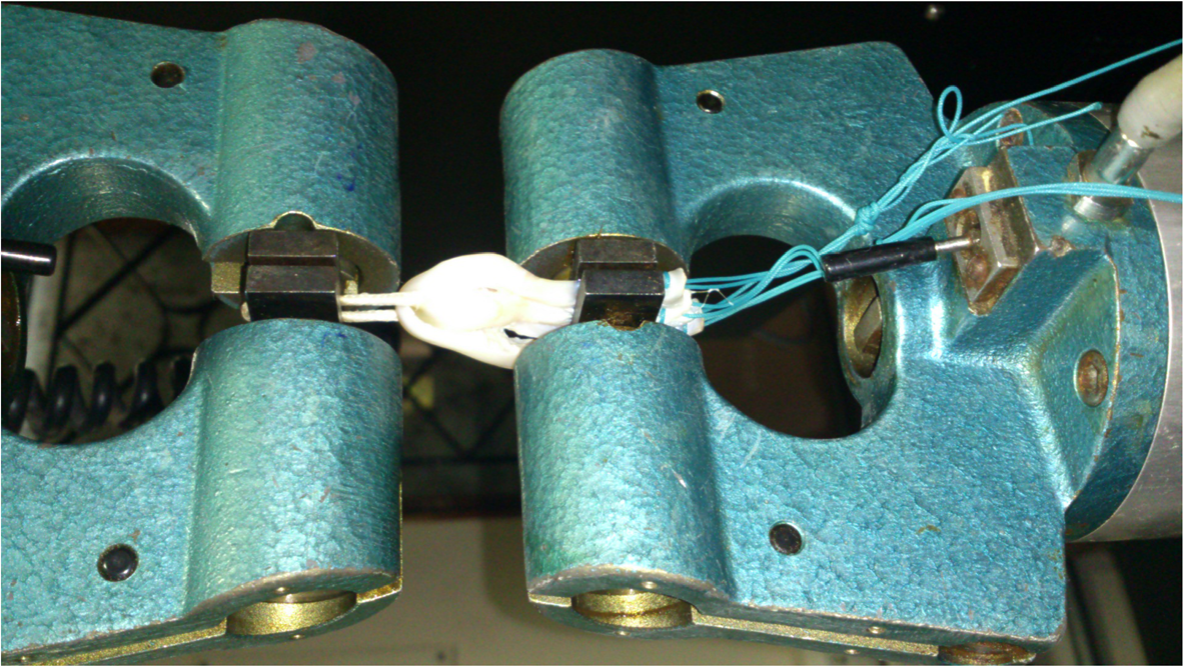
Quadrupled graft construct in test assembly

The specimens were mounted on Material Testing Machine (Instron 6025, Instron Systems, Norwood, Massachusetts). The system consisted of vacuum assisted vices. The button was suspended from the upper vice (to simulate cortical fixation in femur) and the whip-stitched portion of graft was gripped in the lower vice. All strands were evenly gripped in the lower vice (Fig. 5).

The mechanical testing was performed as described by Petre et al. (Petre et al. 2013). The constructs were preloaded from 10 to 50 N at 0.1 Hz for 10 cycles, followed by 1000 cycles of sinusoidal loading between 50 and 250 N at a frequency of 0.5 Hz. After cyclic loading the construct grafts were further displaced at 50 mm/Min until failure. Data were recorded by Instron wave matrix software. The ultimate failure load was recorded by the software at graft slippage or tendon rupture. The displacement after cyclical loading and ultimate load at failure were recorded by the Instron software; site and mechanism of failure were noted by inspection of grafts after mechanical testing. T test was used to assess the statistical significance of the difference between the means.

## Results

**Fig. 6:**
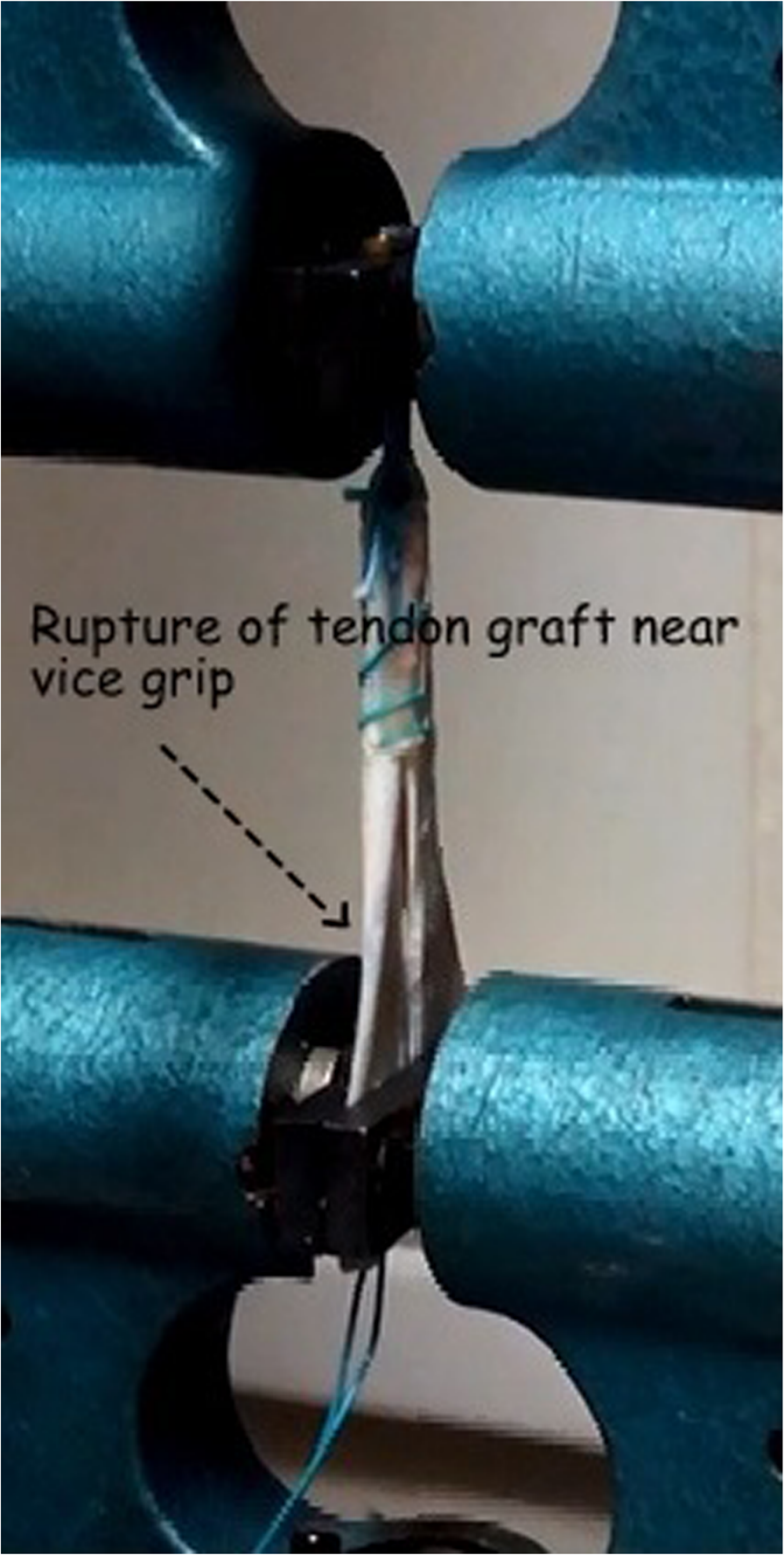
Failure in Group IV, rupture of tendon near the lower vice grip

**Fig. 7:**
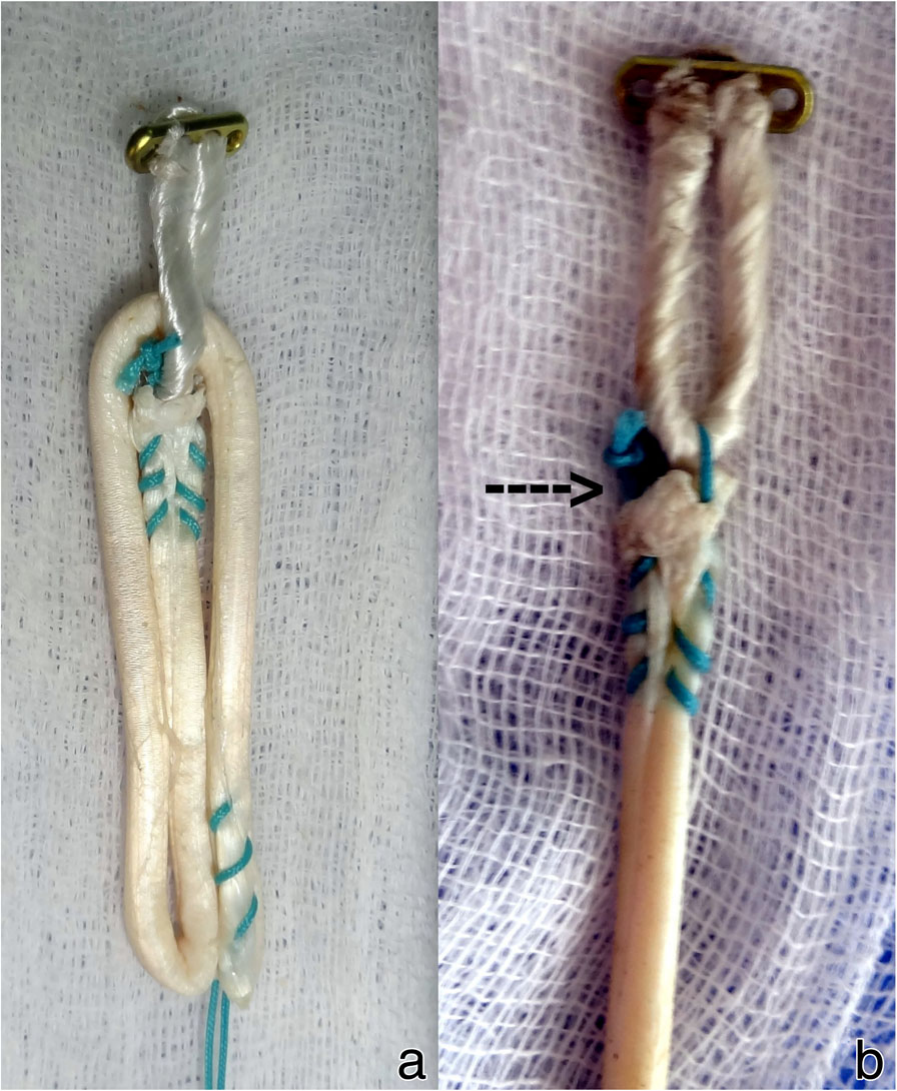
Failure in Group II. **a**. Graft before mechanical testing. **b**. Graft after mechanical testing, arrow pointing to the site of loosening

**Fig. 8:**
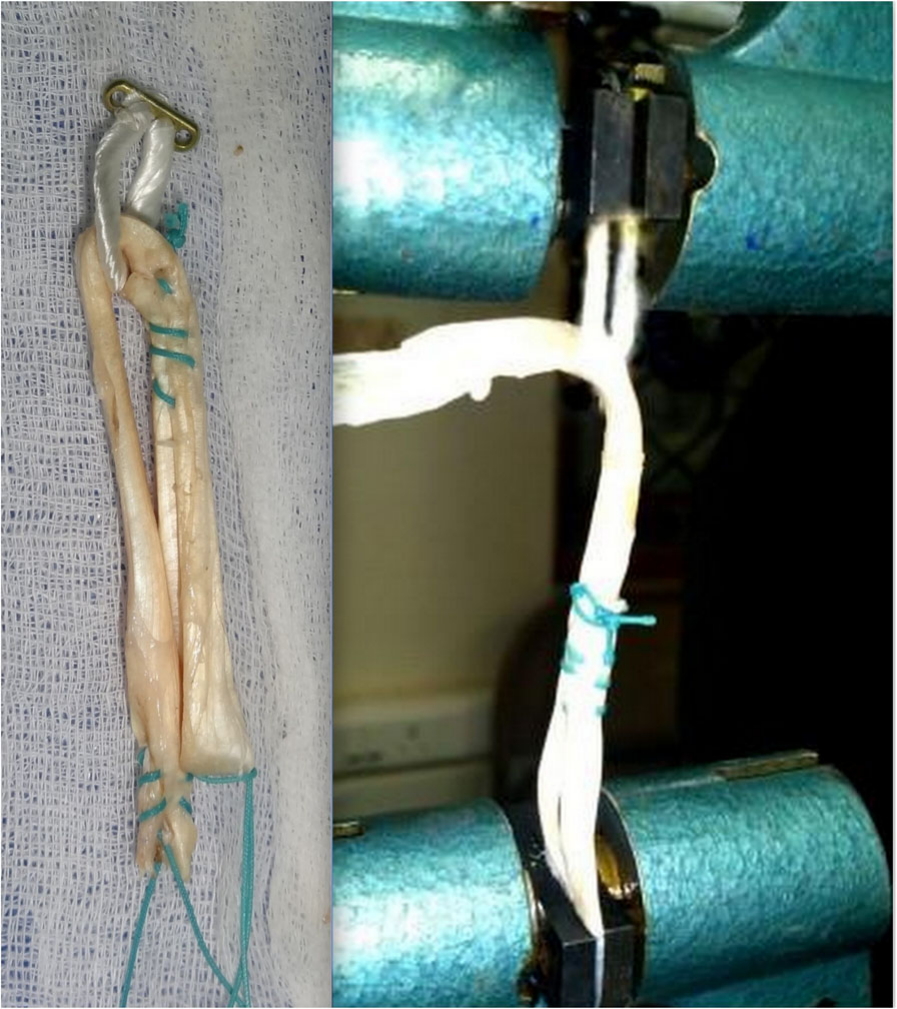
Failure in Group III, Asymmetric loading and early rupture of tendon. (Representative figure)

All samples survived cyclical loading. Group II had the highest displacement under cyclic loading (4.91 ± 0.49 mm). The displacement in the other three groups was 1.13 ± 0.11 mm in Group I, 1.822 ± 0.55 mm in Group III and 1. 126 ± .018 mm in Group IV. The displacement with cyclic loading was significantly more in Group II and Group III (*p* < 0.01). The highest ultimate load at failure (957 ± 23.30 N) was noted in quadrupled tendon (Group I). The lowest load to failure was seen in Group II (590.8 ± 26.55 N). The load to failure values in the other two groups were 682.6 ± 59.28 N in Group III and 963.4 ± 21.72 N in Group IV. The difference in the load at failure was not significant between groups I and IV whereas the groups II and III had statistically significant lesser load to failure than Group I (p < 0.01). All samples in Groups I and IV failed by rupture of tendon near the lower grip (Fig. 6). Samples in Group II failed by pull through of the whipstitch in the central tendon (Fig. 7), while the samples in Group III failed by slippage of graft around the polyester loop of Endobutton CL and consequent asymmetric loading and rupture of single strand of graft (Fig. 8). These results, as well as the graft diameter, length, mode of failure of all the samples are represented in Table 1. Statistical significance for ultimate failure load and displacement after cyclic loading are presented in Table 2.

**Table 1:** Load at failure and the mechanism of failure of all samples

	Sample	Length (cm)	Diameter (mm)	Displacement (mm)	Load at Failure (N)	Mechanism of failure
Group I	1	8	8	1.22	948.06	Tendon rupture
	2	8	8	1.16	966.97	Tendon rupture
	3	8	8	1.07	1003.32	Tendon rupture
	4	8	8	0. 97	975.47	Tendon rupture
	5	8	8	1.36	948.11	Tendon rupture
Group II	1	8	8	4.68	625.01	Pull out of central tendon stitch
	2	8	8	4.49	586.65	Pull out of central tendon stitch
	3	8	8	4.66	616.60	Pull out of central tendon stitch
	4	8	8	4.99	581.14	Pull out of central tendon stitch
	5	8	8	5.72	640.02	Pull out of central tendon stitch
Group III	1	8	8	2.38	649.09	Tendon slip on the loop and tendon rupture
	2	8	8	1.65	649.94	Tendon slip on the loop and tendon rupture
	3	8	8	0.96	660.38	Tendon slip on the loop and tendon rupture
	4	8	8	2.14	738.54	Tendon slip on the loop and tendon rupture
	5	8	8	1.98	778.34	Tendon slip on the loop and tendon rupture
Group IV	1	8	8	1.22	1008.72	Tendon rupture
	2	8	8	0.93	957.42	Tendon rupture
	3	8	8	1.15	968.76	Tendon rupture
	4	8	8	0.97	957.20	Tendon rupture
	5	8	8	1.36	981.28	Tendon rupture -

**Table 2:** Statistical analysis

	Groups	Observed ‘*t*’ value	Table ‘*t*’ value	‘*p*’ value
Displacement	I Vs. II	16.92	3.355 (*p* = 0.01)	*p* < 0.01
	I Vs. III	11.15	3.355 (*p* = 0.01)	*p* < 0.01
	I Vs. IV	0.044	1.806 (*p* = 0.10)	*P* > 0.10
Load at failure	I Vs. II	24.27	3.355 (*p* = 0.01)	*p* < 0.01
	I Vs. III	9.63	3.355 (*p* = 0.01)	*p* < 0.01
	I Vs. IV	0.45	1.806 (*p* = 0.10)	*P* > 0.10

## Discussion

The principal finding of our study is that the tripled tendon grafts have significantly different mechanical properties depending the method of graft preparation. Grafts prepared by suturing three limbs of graft together have equivalent load to failure properties as quadrupled grafts. However, the two other tripled graft constructs tested in on our study had lower load to failure.

Early rehabilitation and ambulation phases after ACL reconstruction entails loads of about 303 to 590 N (Morrison 1970; Shelburne and Pandy 1998, 2002; Shelburne et al. 2004). Of the three tripled graft constructs, groups III and IV had failure loads higher than this value in all the samples.

Cyclic displacement has an effect on a graft’s ability to heal and the long term outcome of graft function. It has been reported that 3.0 mm or more of side-to-side difference in anterior tibial translation, as measured by KT- 1000 arthrometer testing, signifies an ACL failure in nearly all instances (Daniel et al. 1994; Daniel et al. 1985). We measured the displacement with 1000 cycles of loading between 50 N and 250 N following 10 cycles of preloading between 10 N and 50 N. This testing is expected to reproduce the forces on knees following ACL reconstruction, and has been used on previous studies on properties of ACL fixation devices (Petre et al. 2013). Except Group II, other groups had displacement less than 3 mm.

Among the three tripled constructs, one construct, Group II, has been studied earlier. Snow et al. studied the tensile strength and yield load of tripled grafts prepared by securing central tendon to Endobutton loop (Group II in our study) and compared it to doubled grafts (Snow et al. 2012). All samples in their study failed secondary to graft slippage at the tendon screw interface and the authors concluded that there was no mechanical difference in the overall properties between a double tendon and a tripled tendon graft when used in association with suspensory fixation. We noted that the whipstitch placed in the central tendon is pulled out, leading to failure. The difference in modes of failure might due to different tendons used for testing and different test constructs. Flexor tendons, used in the earlier study, were inherently larger than human hamstring tendons (Snow et al. 2012). We used hind limb hoof extensor tendons rather than flexor tendon. Our choice of tendon was guided by the work done by Donahue et al. The authors had compared bovine hoof extensor tendons with human semitendinosous and gracilis grafts and reported that there was no significant difference in the viscoelastic, structural and mechanical behaviour between the two tendons (Donahue et al. 2001). Further, Snow et al. used interference screw to fix the distal end of tendon in a foam block. Hence all the constructs failed at loads consistent with yield load values for interference screw fixation. We gripped the distal end of tendon in the mechanical vice grip of the testing device (Fig. 5) to isolate mechanical properties of graft-Endobutton construct from interference screw fixation. However, in second part of the study, Snow et al. noted notable cyclic elongation occurred in the third limb of tendon (Snow et al. 2012). This finding concurs with the mode of failure noted in our testing. While Snow et al. used Fibrewire or Ultrabraid to stitch the central tendon to the loop of Endobutton, we have used Ethibond. This is unlikely to affect the outcome of this study as all the failures occurred due to pull out of whipstitch from the central tendon rather than by snapping Ethibond thread.

The other two tripled graft constructs tested in our study has not been studied earlier, though they are anecdotal reports of their use (Figueroa et al. 2018; Krishna et al. 2018; Lee 2013; Strobel et al. 2011). Though both the groups III and IV had optimal displacement and load to failure values, there was significant difference in load to failure values between the two groups. Of the three constructs tested, only the Group IV, in which all three strands were tied together, near Endobutton CL loop, had equivalent mechanical properties as quadrupled grafts.

Samples in group II failed by pull out of whipstitch from the central tendon. Samples in the groups I, III, and IV failed by intratendinous stretch and rupture. The samples in group III behaved as doubled tendon graft, with asymmetric strands (Fig. 2). Consequently, the stresses in two strands are unequal, leading to early stretching and failure of one of the strands (Fig. 8). The samples in Group I and Group IV had no asymmetric stress distribution and all the four or three strands, respectively, failed together. This was reflected in the failure loads noted with the four groups.

One limitation of our study is that we have not analysed the differential loading in each of the loop. Snow et al. have noted that the third limb of the tripled tendon undergoes cyclic elongation relative to the doubled portion of the grafts (Snow et al. 2012). Secondly, we have tested the longitudinal load to failure and this represents the extreme case scenario and forces placed on an in vivo graft are likely to be lesser magnitude. In the clinical setting, when the usually experienced forces may be lesser, the differences may not be clinically significant. Furthermore, unidirectional tensile testing to failure does not stimulate the true geometry and loading of the intact ACL. As the knee joint travels through its range of motion, the relative loading in the strands of the ACL and graft changes, and reproducing this was not possible in our study. The third limitation is that we used a clamp on the tibial side whereas in tibial fixation is commonly performed with interferential screw. Hence the mode of failure in clinical setting, when tibial fixation is performed with interferential screw or cortical fixation, may not be the same as noted in the laboratory. Furthermore, in a similar study, Snow et al. identified failure by pull out from the tibial tunnel and this occurred at approximately 565 N–601 N. Historically, tibial fixation of ACL grafts has been considered the weak link of ACL reconstruction (Steiner et al. 1994). The fourth limitation is that we have studied only the load to failure and displacement. Other factors like stiffness, strain, and graft incorporation have not been studied.

We have studied only five samples in each group. The small number samples is a limitation of the study. However the uniform mode of failure in all samples in each group correlates with the statistical observations of the study.

This is only a laboratory study using bovine tendons, using mechanical simulation of worst case scenario following cruciate ligament reconstruction. Therefore, the conclusions that can be drawn are limited. A prospective clinical trial is required comparing these techniques with each other.

## Conclusions

In this mechanical study, we found mechanical differences between the tripled tendons prepared by different methods. Notably, only one method of graft tripling - in which all three strands where sutured together near the loop of suspensory device, had are equivalent mechanical properties as quadrupled graft.
